# The Factors Contributing to Physicians’ Current Use of and Satisfaction With Electronic Health Records in Kuwait’s Public Health Care: Cross-sectional Questionnaire Study

**DOI:** 10.2196/36313

**Published:** 2022-10-07

**Authors:** Jawaher Al-Otaibi, Eleni Tolma, Walid Alali, Dari Alhuwail, Syed Mohamed Aljunid

**Affiliations:** 1 Department of Health Policy and Management College of Public Health Kuwait University Kuwait City Kuwait; 2 Social Behavioral Sciences College of Public Health Kuwait Unviersity Kuwait City Kuwait; 3 Department of Education European University Cyprus Nicosia city Cyprus; 4 College of Life Sciences Kuwait University Kuwait City Kuwait

**Keywords:** health informatics, information systems adoption, electronic health record, EHR, public health informatics

## Abstract

**Background:**

Electronic health record (EHR) has emerged as a backbone health care organization that aims to integrate health care records and automate clinical workflow. With the adoption of the eHealth care system, health information communication technologies and EHRs are offering significant health care advantages in the form of error reduction, improved communication, and patient satisfaction.

**Objective:**

This study aimed to (1) investigate factors associated with physicians’ EHR adoption status and prevalence of EHRs in Kuwait and (2) identify factors predicting physician satisfaction with EHRs in public hospitals in Kuwait.

**Methods:**

This study was conducted at Kuwait’s public Al-Jahra hospital from May to September 2019, using quantitative research methods. Primary data were gathered via questionnaires distributed among 295 physicians recruited using convenience sampling. Data were analyzed in SPSS using descriptive, bivariate, and multivariate linear regression, adjusted for demographics.

**Results:**

Results of the study revealed that the controlled variable of gender (*β*=–.197; *P*=.02) along with explanatory variables, such as training quality (*β*=.068; *P*=.005), perception of barriers (*β*=–.107; *P*=.04), and effect on physician (*β*=.521; *P*<.001) have a significant statistical relationship with physicians’ EHR adoption status. Furthermore, findings also suggested that controlled variables of gender (*β*=–.193; *P*=.02), education (*β*=–.164; *P*=.03), effect on physician (*β*=.417; *P*<.001), and level of ease of use (*β*=.254; *P*<.001) are significant predictors of the degree of physician satisfaction with the EHR system.

**Conclusions:**

The findings of this study had significant managerial and practical implications for creating an inductive environment for the acceptance of EHR systems across a broad spectrum of health care system in Kuwait.

## Introduction

Electronic health record (EHR) systems can provide physicians with accurate information to serve patients more efficiently as compared with paper-based systems [1]. A recent literature review indicated that many health care organizations worldwide, especially in low-income countries, still rely on paper-based systems for maintaining patient records [2]. Research suggests that primary issues faced by traditional systems (ie, paper-based systems) are inaccuracy of information, loss of data, and difficulty in sharing information [3]. In Kuwait, many attempts were made to automate clinical workflows in public hospitals. However, lack of organizational readiness and technical knowledge of the user are primary reasons for EHR implementation failure in Kuwait [4].

Evidence of EHR implementation in public and private health care systems suggests that EHRs are more efficient than paper-based electronic record systems [5]. EHRs significantly improve safety, efficiency, and quality of care provided to patients [6,7].

Furthermore, EHR has a significant impact on the performance of health care workers [6]. An EHR system is an integral part of the clinical decision support system, which provides data to a wide range of health care workers and promptly assists in decisions related to diagnosis and treatment, test results, and the cost of health care [7]. Physicians’ efficient use of EHR can decrease medical errors and provide every health care professional with accurate and timely information [8]. Health care workers can access information quickly and efficiently through the EHR system, which aids in diagnoses and follow-up treatments [9,10]. EHR covers various types of information, from patient medical history to assimilated information from laboratories, specialists, pharmacists, and insurance companies. The EHR system is not only confined to inpatient care but also extends to aftercare with local general practitioners [11].

In contrast, electronic medical record (EMR) refers to the electronic chart of a patient’s medical history assessed by the concerned medical staff. Integration of new technologies, such as Internet of Things, machine learning, artificial intelligence, and decision support, into the electronic health care system module and their implementation has transformed health care. Transformation of traditional data center–based solutions into cloud systems have opened new horizons for applying big data, machine learning, and artificial intelligence [12].

Acceptance of EHR use among physicians in public health care institutes requires considerable investment in training and development. Implementing an EHR system is an issue of change management due to its impact on holistic health care [8]. Thus, the issue of EHR adoption status among physicians has become a significant concern for many public health care institutes, as lack of tech savviness, workflow design, and training are substantial barriers to achieving EHR adoption and satisfaction among physicians [13]

Kuwait provides high-standard health care coverage to its residents. In governmental facilities, free medical treatment is offered to all Kuwaiti nationals. In contrast, foreign residents must pay an annual fee and nominal charges at every visit to access public health care facilities. Kuwait’s government spends 4.6% of its gross domestic product on public health expenditures. Kuwait’s health care sectors accounted for 11% of the public spending of Kuwait in 2018. There are currently 97 primary health care centers in Kuwait overseen by the Ministry of Health [14].

The history of EMR in Kuwait dates back to 2000, when the Ministry of Health introduced a national EMR system across the entire primary care facilities and hospitals. Moreover, in 2013, a national eHealth strategy was launched that attempted to consolidate all patient health records into a single health record file managed by Kuwait’s Ministry of Health and the department of Information Systems [11].

Evidence from a study by Alnashmi et al [15] in Kuwait has shown that most physicians in primary health care settings favor from using the EHR system; however, they suggested additional functionality improvements through digital signatures, integration with artificial intelligence, data warehousing, and big data analytics to enhance the quality of care offered by health care institutes across the country.

Therefore, this research aimed to identify the EHR system adoption status at Al-Jahra hospital in Kuwait, a public hospital operated by the Ministry of Health offering 1234 beds aided by a surgical suite, emergency services, diagnostic center, and outpatient service. It also aimed to measure physicians’ satisfaction with the EHR system by using the factors influencing their satisfaction. The study’s findings posed significant implications for the public health care system in Kuwait to promote greater use of EHR, which can lead to a decrease in medical errors, better health care services, and overall health care cost reduction [8]. Furthermore, digital transformation is the foremost influential agenda for Kuwait Vision 2035 to strengthen investment in high-quality health care and increase the efficiency of the existing health care system [15].

Recent studies [6,7,9] have focused on the theoretical elements predicting EHR adoption among physicians and satisfaction with the existing EHR system. However, there is limited literature on the theoretical aspects that empirically explain the phenomenon of satisfaction with the use of the EHR system [7]. Therefore, this study mainly aimed to fill the research gap by answering critical questions associated with EHR adoption and the degree of physician satisfaction with the existing EHR system used at Al-Jahra hospital. The study aimed to achieve the following research objectives:

- To investigate the prevalence of EHR and EHR adoption status among physicians at Al-Jahra public hospital.
- To investigate the level of satisfaction with EHR use among physicians working at Al-Jahra hospital.
- To investigate factors predicting physician satisfaction with the EHR system at Al-Jahra hospital.

**Figure 1:**
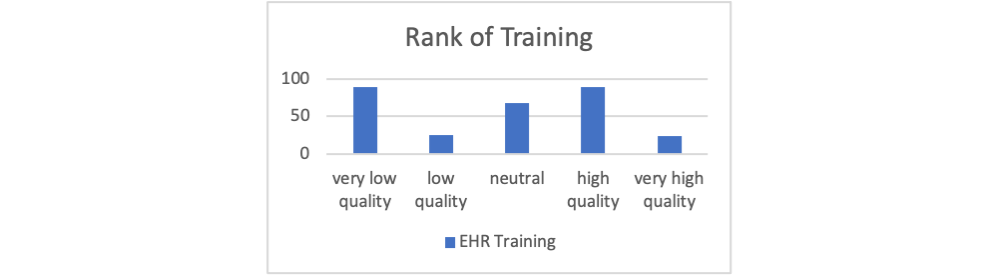
Perception of training received by physicians at Al-Jahra hospital. EHR: electronic health record.

## Methods

### Setting

This cross-sectional study occurred at Al-Jahra hospital, a general public hospital in Kuwait. Data were collected from May 2019 to September 2019. The sample size was selected using the Raosoft calculator, which suggests that the sample size for the population of staff members working at Al-Jahra hospital requires 295 responses [16]. The researcher incorporated convenience sampling methods to recruit participants from various stratified groups (ie, gynecology, general physicians, urologists, orthopedics, ENTs, psychiatry, radiology, pathology, cardiology, and gastroenterology).

### Theoretical Framework

The Technology Acceptance Model (TAM), an information system theory based on the acceptance and use of technology by people, was used to develop the conceptual framework. TAM provides information on a technology-based framework for understanding the user’s adoption of technology and preference for using the advanced technologies, particularly in the workplace environment [6]. The theory is based on the following two primary factors: perceived ease of use and perceived usefulness of technology.

The TAM and the Unified Theory of Acceptance and Use of Technology (UTAUT) are two popular theories used in explaining the use of EHRs; UTAUT helps gauge the degree of physician satisfaction with EHR, as satisfaction is an antecedent of repeated behavioral intention [17].

### Survey Assessment Tool

The survey tool was designed and refined, followed by the pilot testing procedure. The questions included in the survey were based on the information extracted from the literature review. The survey was pilot tested among 33 physicians who had experience using the EHR for clarity, readability, and feedback. The instrument questionnaire reported overall reliability of all items, with α=.886, which suggests that the instrument exhibits high internal consistency.

The final version of the survey consisted of 9 sections and 46 items or questions. They were scored on a 5-point Likert response scale ranging from strongly agree to strongly disagree.

The survey was translated into Arabic and then back-translated into English. An expert (DA) also reviewed the survey in Health Information Systems in Kuwait to ensure cultural and contextual fit.

The survey’s psychometric properties were established using the Confirmatory Factor Analysis (CFA) [18]. The results of the CFA showed that all items in each construct were retained, with the exception of 2 items in the scale ‘Perception of Barriers to Using EHR.’ The final instrument consisted of 8 main variables and 35 items. All scales were reliable, with the lowest reliability score of 0.717 (for ‘Perception of Barriers to Using EHR’) and the highest score of 0.897 (for ‘Level of Ease of EHR Function’). There were 2 dependent variables. The first was the physician EHR adoption status, and the second was the degree of physician satisfaction with EHR. The independent variables, as directed by the TAM model, include satisfaction with technical support, preference for using a new EHR system, preference to go back to a paper-based system, perception of barriers to using EHR, the effect of the use of EHR on physician, and level of ease of EHR. The demographics measured in the survey were gender, nationality, age group, education, years of experience, work department, and job title. The quality of the related training was also added as an independent variable based on the theocratical insight of the TAM model, which suggests that training paradigms significantly influence behavioral intentions [19].

### Inclusion and Exclusion Criteria

The target population consisted of physicians working at Al-Jahra hospital in Kuwait. Inclusion criteria involved (1) employees of Al-Jahra hospital, (2) physicians, and (3) experience using the EHR system in the hospital, whereas exclusion criteria included (1) former employees of Al-Jahra hospital, (2) administrative staff, (3) nurses, (4) technicians, and (5) physicians working with Al-Jahra on a contractual agreement.

### Population and Sampling

According to a previous study, 55% of the physicians in Kuwait are already using an existing adopt EHR system [20]. Considering this adoption rate, a finite population size of 503, a 5% error rate, and a design effect of 1, the required sample consisted of 217 research participants. Assuming a nonresponse rate of 20%, a target sample size of 277 physicians was required for the quantitative study.

### Ethics Approval

Ethical approval (2019/1093) was obtained from the Kuwait Ministry of Health Ethical Committee. All research participants signed the informed consent form, which clearly stated the study’s purposes, data use, and participants’ safety (ie, confidentiality and anonymity). 

### Statistical Analysis

The paper survey was self-administered. The response rate was 95%. Missing values were treated in SPSS using missing values analysis, which suggested that missing values were completely at random, and there was no pattern that resulted in the pairwise deletion of data.

The data were analyzed using the IBM Statistical Package for Social Sciences (version 23; IBM Corp) [21]. Descriptive statistics analysis was also conducted, followed by bivariate analysis. The most common test used in the bivariate analysis was the Pearson correlation analysis. The final statistical analysis used was multiple regression analysis to test the contribution of the independent variables to the dependent variables (ie, current use of EHR and satisfaction with EHRs), adjusted for the demographics. This was done in two steps; first, in model 1, only demographic variables were added to the analysis; then, in model 2, both demographics and the independent variables were added. The alpha level set for this study was .05.

## Results

### Descriptive Statistics

Of 295 participants, the majority of the participants were male (n=242, 82%) and non-Kuwaitis, (n=259, 88%) from India, Egypt, Asia, Africa, and other parts of the Middle East and North Africa (or MENA) region. Most of the respondents were generally young (n=120, 40.7%), between 30 and 39 years of age, and were experienced physicians (n=100, 33.9% had 5-10 years of work experience). Most of them were registrars (n=88, 29.8%) and gynecologists (n=114, 38.2%), as shown in Table S1 in Multimedia Appendix 1.

In terms of behavioral characteristics, almost 2 of 5 of the respondents (n=124, 42%) reported using the EHR system for more than 5 years. There was a lack of consensus among respondents regarding the quality of related training received; for example, 89 (30.2%) reported receiving low-quality training, whereas another 89 (30.2%) reported receiving high-quality training on EHR system use.

### Bivariate Correlation

Regarding bivariate analysis, the degree of physician satisfaction with the EHR system is strongly correlated with the preference for using the new EHR system (*r*=0.797) and its effect on physician (*r*=0.744); it was moderately correlated with satisfaction with technical support (*r*=0.632) and level of ease of EHR system use (*r*=0.698), as shown in Tables S2 and S3 in Multimedia Appendix 1.

### Multiple Regression Analysis

The first series of multiple regression analyses that included all independent variables in the prediction of the EHR adoption status and adjusted for demographic variables showed that the perception of barriers (*β*=–.0107; *P*=.04), the effect of the use of EHR on physician (*β*=.521; *P*<.001), and training quality (*β*=.068; *P*=.005) are significant predictors of physician EHR adoption status (*R*^2^=0.56), as shown in Table S4 in Multimedia Appendix 1.

In the second series of multiple regression analyses that included all independent variables in the prediction of the degree of satisfaction with EHR use and adjusted for demographic variables, findings showed that gender (*β*=–.1931; *P*=.02), education (*β*=–.164; *P*=.03), effect on physician (*β*=.417; *P*<.001), and level of ease of EHR use (*β*=.254; *P*<.001) are significant predictors of the degree of physician satisfaction with the EHR system (*R*^2^=0.62), as shown in Table S5 in Multimedia Appendix 1.

## Discussion

### Principal Findings

The study’s primary purpose was to examine the psychosocial factors associated with physicians’ use of EHR and satisfaction with the EHR system at Al-Jahra public hospital in Kuwait. Findings of the study show that the level of EHR adaption status can be predicted with the controlled variable of gender along with explanatory variables, that is, training quality, perception of barriers to using EHR, and effect on the physician. Furthermore, findings also suggested that controlled variables (ie, gender and education) along with explanatory variables (ie, effect on physicians and level of ease of EHR system) significantly influence physician EHR adoption status. The gender of the physician can also play an important role in the use of EHR. In our study, females were more likely than males to use the EHR system and were more satisfied with it, as supported by the literature [22].

The study’s findings validate previous studies [12], which highlight the role of risk and trust relationship in predicting EHR adoption status, as findings revealed that the performance and trust relationship implied by the UTAUT model had no impact on physician intention to use an EHR system. This implies that developers, marketers, and medical professionals should improve and optimize patient communication in the EHR system. Our findings validate previous evidence [21] and also suggest that social factors have a negligible effect on physician intention to adopt EHR system, as physicians are driven by their attitudes, ability to control innovation offered by the EHR system, and holistic benefits offered by the system. Findings also validate the role of training in influencing EHR system adoption status among physicians, as evidence from a study by Dunton [23] suggests that training influences perceived usefulness and perceived ease of use as well as external factors, which significantly enhance physician EHR system adoption status.

In terms of the prediction of EHR use, the most important factor was the effect that the use of EHR had on physicians’ work. This implies that physicians will be more inclined toward using the EHR system if they perceive a beneficial effect of the use of EHR on their work. In addition, the length of use of EHR also had a positive contribution to the prediction of EHR use. This is not surprising, since using the EHR system for an extended period will lead to adopting the EHR system, according to a study by Liang et al [20].

Regarding the prediction of physicians’ satisfaction with the use of EHR, the most significant contributor was the effect of EHR use on physicians’ work, as supported by the findings of a previous study [24]. Specifically, it was found that the higher the perceptions of the positive effects of EHR on physicians’ work, the more likely it will be for the physicians to be satisfied with the use of the EHR system. The second most important contributor was the degree of ease of EHR use. Consistent with the findings of other studies, as physicians start to experience the ease of using the EHR system, they will start adopting the EHR system [3]. Moreover, another study [25] found that perceived usefulness and perceived ease of use increase the acceptance of using the EHR system and hence the satisfaction with it. As it was explained, accepting the use of the EHR system was an indication of the level of satisfaction of the physicians. Therefore, it was not surprising that this study found the degree of ease of using EHR as an important factor in satisfaction with it. In other words, as physicians perceived the EHR system to be easy to use, they were more likely to use it and experience higher satisfaction levels.

Another unique finding concerning the satisfaction with EHR was related to the academic background of participants. According to the results, this characteristic was a significant factor. This implies that the knowledge and academic experience of the physicians might have an impact on their satisfaction level. Those physicians with higher qualifications will tend to be more satisfied with the EHR system compared with others because they might believe that using the EHR system would allow them to serve the patients better. Evidence from a study [26] demonstrated that physicians with higher levels of education had higher levels of satisfaction with EHR use.

Finally, age was also significant in the prediction of EHR use but in a negative way. Older physicians were more reluctant to use the EHR system, as supported by a previous study [17]. In our study, the demographics were treated as controlled variables. Therefore, their effect on the regression model and other variables was neglected.

From a programmatic perspective, the following are some recommendations for public health professionals in their effort to promote the use of EHR and increase satisfaction with EHR among those who are already using it in governmental hospitals of Kuwait:

- Professionals should first conduct a needs assessment, identify perceived barriers among physicians, and try to address those barriers.
- Public health professionals should focus on improving the functionality of the EHR system and make it as easy as possible to operate; this will encourage physicians to use it more often and rely on the EHR system when seeing patients.
- Public health professionals are advised to emphasize promoting the EHR system’s positive effects on physicians’ work, which could be done through health communication campaigns.

### Limitations

There were some limitations to the study. The results of this study were only limited to Al-Jahra hospital, where the study took place. However, this study can be generalized, and the outcomes can be easily applied to other public sector hospitals in Kuwait, as the research examined satisfaction with EHR and adoption of the EHR system. Second, the TAM was used to assess the adoption of EHR and satisfaction with it. However, the TAM and UTUAT models were not fully applied to this study, as the attitude was not examined. Due to the questionnaire being long, the decision was made to shorten it, so more physicians were interested in filling out the questionnaire and participating in the study. The study did not use purposive sampling as convenience sampling for data collection. The use of a random sample might have produced a different result. The findings of the study are not generalizable to all hospitals in Kuwait. Thus, more studies need to be conducted to validate whether other public hospitals exhibit the same phenomenon.

### Future Research

Since this study could not cover all aspects that might be useful in examining the satisfaction with EHR system and current use of it, the following future studies must be carried out. First, the theoretical framework should be expanded to include physicians’ attitudes toward using EHR. Research should be conducted that fully uses the TAM by including physicians’ attitudes. It might prove important to examine physicians’ attitudes, since some physicians might have a positive predisposition toward using EHR but still not use it. It would be interesting from a theoretical and programmatic perspective to examine how attitude relates to intention by itself. Second, a follow-up qualitative study through several interviews with senior physicians and hospital officials should be conducted. Such a study will help identify more in-depth information behind using or not using the EHR system. For instance, qualitative research can complement quantitative research results and help us discover the perceived barriers to adopting the EHR system. In addition, qualitative research can help us find answers to surprising results, such as the fact that women are more likely to adopt an EHR system. This study can be replicated in other governmental hospitals in Kuwait to reach a better understanding of how prevalent the use of EHR is and the degree of satisfaction with its use.

### Conclusions

There are important takeaways from the results of this study. First, there is still a need to further expand the EHR system adoption at Al-Jahra hospital, since almost 1 in 5 physicians has never used EHR or has used EHR for less than a year. This could be justified as they may have joined the hospital recently. Second, to increase the adoption rate and satisfaction with the current use of EHR among physicians, public health professionals can make the benefits of EHR adoption more visible to the physicians, remove perceived barriers, make the use of the EHR system as easy as possible, and incorporate a high-quality related training, while providing continuous technical support. Results from this study can be helpful to other governmental hospitals in Kuwait in their efforts to enhance the levels of adoption and satisfaction with the EHR system. The EHR system has many benefits, and it can be fully realized only when all physicians in governmental hospitals in Kuwait fully adopt it.

## Appendix

Multimedia Appendix 1 Tables S1-S5.

## Abbreviations

EHR: electronic health record
EMR: electronic medical record
TAM: Technology Acceptance Model
UTAUT: Unified Theory of Acceptance and Use of Technology
