# Supplementary material for: The Factors Contributing to Physicians’ Current Use of and Satisfaction With Electronic Health Records in Kuwait’s Public Health Care: Cross-sectional Questionnaire Study
Source: JMIR Med Inform. 2022 Oct 7;10(10):e36313. doi: 10.2196/36313 (PMC9587489; doi:10.2196/36313)
Supplement: Multimedia Appendix 1 [file medinform_v10i10e36313_app1.docx]

**Multimedia Appendix 1**

Table S1 Appendix 1‏

| Characteristics | | Participants |
| --- | --- | --- |
| Gender | |  |
|  | Male | 242 (82) |
|  | Female | 53 (18) |
| Nationality | | |
|  | Kuwaiti | 36 (12.2) |
|  | Non-Kuwaiti | 259 (87.8) |
| Age Group | | |
|  | 20-29 | 25 (8.5) |
|  | 30-39 | 120 (40.7) |
|  | 40-49 | 100 (33.9) |
|  | 50-59 | 50 (16.9) |
| Education | | |
|  | Diploma | 16 (5.4) |
|  | Bachelor (BA) | 23 (7.8) |
|  | Masters (MA) | 166 (56.3) |
|  | PhD | 71 (24.1) |
|  | Medical Council | 16 (5.4) |
|  | Others | 3 (1) |
| Years of Experience | | |
|  | Less than 5 | 50 (16.9) |
|  | 5-10 years | 100 (33.9) |
|  | 11-20 years | 96 (32.5) |
|  | 21-30 years | 36 (12.2) |
|  | Over 30 years | 13 (4.4) |
| Work Department | | |
|  | Internal Medicine | 93 (31.5) |
|  | Surgery | 26 (8.8) |
|  | Orthopaedics | 15 (5.1) |
|  | Otorhinolaryngology | 27 (9.2) |
|  | Gynaecology | 114 (38.2) |
|  | Paediatrics | 1 (0.3) |
|  | Anesthesiology | 1 (0.3) |
|  | Nuclear Medicine | 7 (2.4) |
|  | Natural Medicine | 4 (1.4) |
|  | Accidents | 7 (2.4) |
| Job Title | | |
|  | Resident | 12 (4.1) |
|  | Assistant Registrar | 25 (8.5) |
|  | First Registrar | 60 (20.3) |
|  | Registrar | 88 (29.8) |
|  | Senior Registrar | 44 (14.9) |
|  | Specialist | 23 (7.8) |
|  | First Specialist | 16 (5.4) |
|  | Consultant | 23 (7.8) |
|  | Others | 4 (1.4) |

Table S2 Appendix 1‏

|  | Variable | Satisfaction with Technical Support | Preference of using New EHR System | Preference to go Back to Paper Based system | Perception of Barriers to using EHR |
| --- | --- | --- | --- | --- | --- |
| Satisfaction with Technical Support | | | | | |
|  | r | 1 | .791^**^ | .107 | -.290^**^ |
|  | *P* Value | .000 | .067 | .000 | .000 |
| Preference of using New EHR System | | | | | |
|  | r | .791^**^ | 1 | -.063 | -.347^**^ |
|  | *P* Value | .000 |  | .282 | .000 |
| Preference to go Back to Paper Based system | | | | | |
|  | r | .107 | -.063 | 1 | -.107 |
|  | *P* Value | .067 | .282 |  | .066 |
| Perception of Barriers to using EHR | | | | | |
|  | r | -.290^**^ | -.347^**^ | -.107 | 1 |
|  | *P* Value | .000 | .000 | .066 |  |
| Degree of Physician Satisfaction with EHR | | | | | |
|  | r | .632^**^ | .797^**^ | -.049 | -.398^**^ |
|  | *P* Value | .000 | .000 | .400 | .000 |
| Physician EHR Adaption Status | | | | | |
|  | r | .580^**^ | .586^**^ | -.049 | -.406^**^ |
|  | *P* Value | .000 | .000 | .404 | .000 |
| Effect on Physician | | | | | |
|  | r | .613^**^ | .672^**^ | -.116^*^ | -.441^**^ |
|  | *P* Value | .000 | .000 | .046 | .000 |
| Level of Ease of EHR | | | | | |
|  | r | .588^**^ | .719^**^ | -.127^*^ | -.385^**^ |
|  | *P* Value | .000 | .000 | .029 | .000 |

Table S3 Appendix 1‏

|  | Variable | Satisfaction with Technical Support | Preference of using New EHR System | Preference to go Back to Paper Based system | Perception of Barriers to using EHR |
| --- | --- | --- | --- | --- | --- |
| Satisfaction with Technical Support | | | | | |
|  | r | .632^**^ | .580^**^ | .613^**^ | .588^**^ |
|  | *P* Value | .000 | .000 | .000 | .000 |
| Preference of using New EHR System | | | | | |
|  | r | .797^**^ | .586^**^ | .672^**^ | .719^**^ |
|  | *P* Value | .000 | .000 | .000 | .000 |
| Preference to go Back to Paper Based system | | | | | |
|  | r | -.049 | -.049 | -.116^*^ | -.127^*^ |
|  | *P* Value | .400 | .404 | .046 | .029 |
| Perception of Barriers to using EHR | | | | | |
|  | r | -.398^**^ | -.406^**^ | -.441^**^ | -.385^**^ |
|  | *P* Value | .000 | .000 | .000 | .000 |
| Degree of Physician Satisfaction with EHR | | | | | |
|  | r | 1 | .675^**^ | .744^**^ | .698^**^ |
|  | *P* Value |  | .000 | .000 | .000 |
| Physician EHR Adaption Status | | | | | |
|  | r | .675^**^ | 1 | .711^**^ | .609^**^ |
|  | *P* Value | .000 |  | .000 | .000 |
| Effect on Physician | | | | | |
|  | r | .744^**^ | .711^**^ | 1 | .778^**^ |
|  | *P* Value | .000 | .000 |  | .000 |
| Level of Ease of EHR | | | | | |
|  | r | .698^**^ | .609^**^ | .778^**^ | 1 |
|  | *P* Value | .000 | .000 | .000 |  |

Table S4 Appendix 1‏

| **Coefficients** | | | | | | |
| --- | --- | --- | --- | --- | --- | --- |
| Model | | Unstandardized Coefficients | | Standardized Coefficients | t | Sig. |
|  |  | B | Std. Error | Beta |  |  |
| 1 | (Constant) | 1.497 | .340 |  | 4.408 | .000 |
|  | Age | -.102 | .070 | -.067 | -1.467 | .143 |
|  | Gender | -.197 | .087 | -.100 | -2.269 | .024 |
|  | Nationality | .167 | .107 | .072 | 1.557 | .121 |
|  | Education | .115 | .074 | .070 | 1.538 | .125 |
|  | Experience | .032 | .100 | .016 | .318 | .751 |
|  | Use | -.055 | .067 | -.036 | -.820 | .413 |
|  | Training quality | .068 | .024 | .123 | 2.848 | .005 |
|  | Perception of Barriers to use EHR | -.107 | .053 | -.094 | -2.022 | .044 |
|  | Effect on Physicians | .521 | .059 | .647 | 8.892 | .000 |
|  | Level of Ease of EHR | .053 | .056 | .065 | .946 | .345 |

| a. Dependent Variable: Physician HER adaption Status |
| --- |

Table S5 Appendix 1‏

| **Coefficients** | | | | | | |
| --- | --- | --- | --- | --- | --- | --- |
| Model | | Unstandardized Coefficients | | Standardized Coefficients | t | Sig. |
|  |  | B | Std. Error | Beta |  |  |
| 1 | (Constant) | 2.277 | .334 |  | 6.819 | .000 |
|  | Age | -.103 | .069 | -.063 | -1.500 | .135 |
|  | Gender | -.193 | .085 | -.091 | -2.265 | .024 |
|  | Nationality | -.153 | .106 | -.062 | -1.446 | .149 |
|  | Education | -.164 | .073 | -.093 | -2.242 | .026 |
|  | Experience | -.161 | .098 | -.074 | -1.634 | .103 |
|  | Use | .070 | .065 | .043 | 1.075 | .283 |
|  | Training quality | .030 | .024 | .051 | 1.287 | .199 |
|  | Perception of Barrier to use EHR | -.084 | .052 | -.068 | -1.609 | .109 |
|  | Effect on Physicians | .417 | .058 | .485 | 7.245 | .000 |
|  | Level of Ease of EHR | .254 | .056 | .291 | 4.579 | .000 |

| a. Dependent Variable: Degree of Physician Satisfaction with EHR |
| --- |

Table Appendix 1. 6: Gap Analysis

| **ANNEX** | | | | | | | |
| --- | --- | --- | --- | --- | --- | --- | --- |
| **No** | **Aspect** | **Questions** | **Yes** | **No** | **Evidence** | **Non-Conformity** | **Gap** |
| **A.1** | **Barrier to Use of EHR System** | | | | | | |
| **What are the barriers to using EHR systems at Public hospitals amongst physicians?** | | | | | | | |
| **A1.1** | **Learning Constraints** | **Were physicians at Al-Jahra handy and had enough knowledge of using Information system?** |  | **X** | **Many physicians who are older rely on other assistant staff members for data entry and generation of reports from the EHR system** | **Either system is complex or there is no support mechanism at Al-Jahra hospital** | **This leads us to the development of objective to investigate factors influencing EHR adaption status amongst the physicians.** |
| **A.2** | **Satisfaction with EHR system** | | | | | | |
| **What factors affect physician working at public hospital with the EHR system?** | | | | | | | |
| **A.2.1** | **Interdepartmental Workflow integration** | **Does EHR system at Al-Jahra hospital covers all inpatient and outpatient workflows?** |  | **X** | **Many physicians reported lack of complete automation as EHR works in parallel with other system** | **EHR system does not offer holistic picture of automation at Al-Jahra hospital** | **Physicians at Al-Jahra have a tendency to shift to paper-based system due to lack of complete automation which leads us to an objective of factors influencing physician satisfaction with EHR system.** |
| **A.3** | **On-job and Off job User trainings** | | | | | | |
|  | **Training of Employees** | **Do newly hired physicians receive training on usage and utility of system?** |  | **X** | **Hospital relies on on-job training. IT department is only responsible for providing use manuals and create credentials for new joiners** | **No formal training procedure and lack of training quality reported at Al-Jahra hospital** | **This leads to development of objective to investigate the impact of training quality of EHR system level of adaption of physician satisfaction with EHR system.** |
